# Clinical and Radiological Evaluation of a Self-Condensing Bone Implant in One-Stage Sinus Augmentation: A 3-Year Follow-Up Retrospective Study

**DOI:** 10.3390/ijerph20032583

**Published:** 2023-01-31

**Authors:** Luca Comuzzi, Margherita Tumedei, Morena Petrini, Tea Romasco, Felice Lorusso, Francesco De Angelis, Adriano Piattelli, Marco Tatullo, Natalia Di Pietro

**Affiliations:** 1 Independent Researcher, San Vendemiano-Conegliano, 31020 Treviso, Italy; 2 Department of Medical, Surgical, and Dental Sciences, University of Milan, 20122 Milan, Italy; 3 Department of Medical, Oral and Biotechnological Sciences, “G. d’Annunzio” University of Chieti-Pescara, 66013 Chieti, Italy; 4 Center for Advanced Studies and Technology-CAST, “G. d’Annunzio” University of Chieti-Pescara, 66013 Chieti, Italy; 5 School of Dentistry, Saint Camillus International University of Health and Medical Sciences, 00131 Rome, Italy; 6 Facultad de Medicina, UCAM Universidad Católica San Antonio de Murcia, 30107 Murcia, Spain; 7 Department of Translational Biomedicine and Neurosciences (DiBraiN), University of Bari Aldo Moro, 70124 Bari, Italy

**Keywords:** biomaterial, cone-beam computed tomography, maxillary sinus augmentation, xenografts, one-stage sinus augmentation, self-condensing implants

## Abstract

Stabilization of dental implants in the sinus region with a bone height below 4 mm gen-erally requires a two-stage sinus floor elevation surgery. To improve this aspect, the aim of this retrospective study was to demonstrate the feasibility of performing a one-stage maxillary sinus augmentation using an innovative self-condensing implant design, even in case of a bone height close to 2 mm. Clinical and radiological outcomes from 54 patients (26 females; 28 males; 69 total implants positioned) were analyzed 3 years post-surgery. The three-dimensional grafts change was evaluated by Cone-Beam Computed Tomography (CBCT) before surgery (T0), immediately after surgery (T1), and 1-year post-surgery (T2). The sinus floor levels measured at the medial (M-W), middle (MD-W), and lateral (L-W) walls reported: M-W of 1.9 ± 2.4 mm (T1) and 1.7 ± 2.6 mm (T2); MD-W of −0.1 ± 2.7 mm (T1) and 0.7 ± 2.4 mm (T2); L-W of 3.1 ± 3.0 mm (T1) and 3.1 ± 3.0 mm (T2); besides a bone crest height (C-F) of 4.6 ± 2.0 mm (T1) and 12.1 ± 1.4 mm (T2). Moreover, after 3 years only 1 implant was lost, and so an implant survival rate of 98.55% was recorded. In conclusion, these results suggest the efficacy of using this implant design for a one-stage sinus lift approach, not only in terms of increased implant survival rate and decreased marginal bone loss, but also for its potential applicability in case of reduced bone height.

## 1. Introduction

The implant placement in the posterior maxilla region could be considered clinically challenging in case of severe atrophy of the edentulous crestal bone and pneumatization of the maxillary sinus [1,2]. Indeed, the reduced amount and quality of the bone require more technical, sensitive procedures that imply additional biological costs, treatment time, and increased morbidity [3,4]. The bone augmentation of the atrophic maxilla can be performed using a sinus lifting technique, as introduced by Tatum in 1986 [5], that generally requires an healing time of 6–9 months before implant insertion [6].

Different biomaterials are currently used for bone regeneration and, according to their origin, they can be classified in: autologous, homologous, heterologous, and alloplastic [7,8,9,10]. Many reviews assessed the significant advantages of using a combination of different grafting materials during bone augmentation procedures for a better new bone formation, and the scaffold origin seems to be not a determinant criterion for this aim [7,8,11]. Rather, different anatomical factors may influence the new bone formation, especially in the posterior upper maxilla. For example, the healing and mineralization of the bone substitutes are negatively correlated with the width of the bucco-palatal sinus, the width of the bony window during lateral sinus augmentation, and with a reduced crestal height. Moreover, another critical factor that could influence the bone formation and the grafting success is vascularization [12,13].

In the last years, many different surgical techniques have been developed to treat the atrophic maxilla. Sinus grafting and implant placement can be performed simultaneously (one-stage) or in a delayed approach (two-stage). The choice is determined by the amount of the residual crestal bone height (RBH). In case of crestal bone height less than 3–4 mm, a two-stage implant placement is generally indicated with a time interval of 6–8 months between the bone augmentation and the implant insertion [13]. Conversely, crestal native bone height more than 3–4 mm is usually considered sufficient to provide an adequate mechanical stability (>25 Ncm) for the simultaneous implant placement and the sinus elevation [14]. However, several studies reported no statistically significant differences between implants positioned simultaneously with sinus augmentation versus a two-stage technique [12,13].

Primary stability has been recognized as a determinant criterion to achieve an effective bone-implant osseointegration [15]. In fact, the one-stage approach requires a sufficient fixture stability in order to ensure the organization of the bone clot, avoiding micromovements of the implant and improving new bone formation and the graft osseointegration [16].

According to Lundgren et al. [17], two sinus floor elevation approaches can be considered, aiming at increasing the bone height in the posterior maxilla for the insertion and integration of dental implants. A transcrestal sinus floor method is recommended in situations with a sufficient width for the implant placement and a RBH > 5 mm, whereas a lateral sinus floor elevation method is indicated when ≤5-mm height of bone is available and several teeth are to be replaced, with or without the use of grafting materials. Moreover, a one-stage procedure is preferred since a high primary stability and a time-saving in implant placement can be ensured. Recently, a novel self-condensing implant design (Sinus-Plant, Oralplant Suisse, Mendrisio, Switzerland) has been proposed to improve the primary stability of dental implants inserted in the posterior maxilla with a limited residual native bone height [18]. As for other similar implants, this particular tronco-conical morphology allows an optimal bone preservation by creating a compact layer of bone along the surface of the osteotomy and providing a conical coupling between the implant and the bone [19,20]. The narrow threading further increased the bone-implant contact, helping to control the implant progression in low-height bone, limiting peak stress and practicing a controlled lateral osseocondensation. Moreover, the rounded apex with a lapping surface limits the impacting insertion of the implant towards the sinus membrane. Additionally, through this conometric procedure coupling implant and bone, it is possible to combine a safe barrier against bacterial infiltrations reaching an excellent stability, and, at the same time, the Switching Platform connection reduces peri-implant bone resorption cones and allows for the abutments to be easily removed, if necessary.

Based on this evidence, the aim of the present retrospective study was to evaluate the 3-year follow-up clinical and radiographical outcomes of an innovative dental implant design, which could allow to obtain simultaneously (one-stage approach) an increase in sinus maxilla and the implant insertion, even in challenging scenarios, such as with bone heights close to 2 mm.

## 2. Materials and Methods

### 2.1. Study Design

This study was conducted according to the guidelines of the Declaration of Helsinki and the Good Clinical Practice Guidelines.

The present retrospective study included adult patients who spontaneously came to the clinic in need of a partial restoration of the atrophic posterior maxilla. All of them underwent maxillary sinus augmentation procedures, which required the use of different bone grafts, depending on the case and the availability of the product, and implant sizes, according to the position and the residual bone height. Subsequently, patients have been followed for 3 years after implant placement, and data was recorded prior to the surgery (T0), immediately after surgery (T1), after 1 year (T2), and at the end of the 3-year follow-up. Inclusion and exclusion criteria were drawn up as follows:

– Inclusion criteria

- Patients over 18 years of age;
- Patients undergoing a lateral or transcrestal approach of maxillary sinus floor augmentation with simultaneous implant placement;
- Patients in general good health and with no history of systemic diseases or medications that could interfere with the surgical treatment;
- Patients compliant with a supportive maintenance therapy after maxillary sinus floor augmentation procedures.

– Exclusion criteria

- Patients with active infection or disease affecting bone metabolism and wound healing;
- Patients with active oral infection, such as untreated pockets on natural teeth or periapical fistulas;
- Patients who have undergone radiotherapy in the craniofacial region within the past 12 months;
- Patients with systemic diseases that could compromise osseointegration, such as untreated diabetes;
- Patients with a history of maxillary sinusitis or sinus surgery;
- Patients who regularly used steroids or other drugs involved in bone turnover;
- Patients who were pregnant or breastfeeding;
- Patients who smoked more than 10 cigarettes/day.

Surgeries were performed by a single clinical surgeon, Dr. Luca Comuzzi, the first author of this work, in his private dental practice in Conegliano Veneto (TV), Italy.

Each surgery consisted of a one-stage implant placement and a sinus augmentation via the lateral window technique or the transcrestal approach. The choice of the sinus augmentation approach was made on the basis of the preoperative radiographic evaluation that evidenced both the bone quality and quantity. If the RBH ranged from 1.0 to 5.0 mm, a lateral technique was performed. On the contrary, if the RBH was included between 5.1 and 9.0 mm, a transcrestal protocol was applied.

The main characteristics of the study population are described in Table 1.

Consequently, the lateral approach for the sinus augmentation was used to insert 46 implants in 31 patients, and the transcrestal one was performed in 23 patients for a total of 23 implants. Specifically, the study comprehended a total of 69 dental implants named Sinus-Plant (Oralplant Suisse, Mendrisio, Switzerland) with a width of 4.5 mm and a length ranging from 9 to 11 mm, which were inserted in 54 adult patients (26 female; 28 male), according to the implant size and position (Table 2) and to the bone graft used (Table 3).

### 2.2. Implant Characteristics

**Figure 1:**
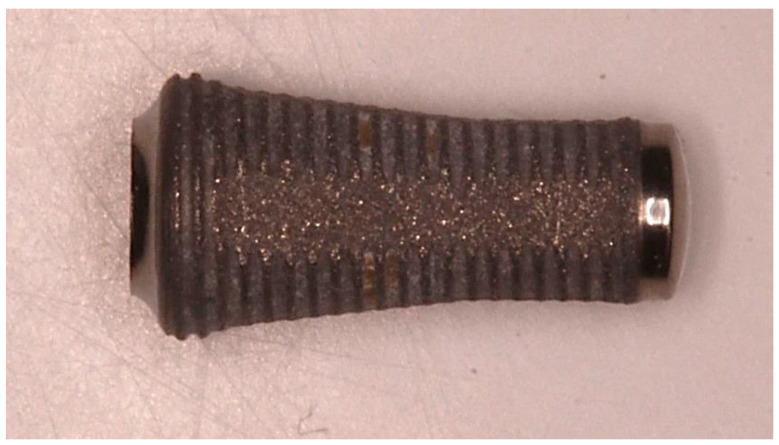
Detail of the Sinus-Plant implants with a troncoconical macro-morphology.

The characteristics of the Sinus-Plant implants (Oralplant Suisse, Mendrisio, Switzerland) have been previously described [20]. Briefly, these implants were characterized by: a tronco-conical shape macromorphology; a titanium lapping surface at the implant rounded apex, able to preserve the Schneiderian membrane integrity; a thread design with a narrow inter-thread distance of 0.4 mm, limiting stress and promoting progressive bone expansion and osseodensification; a Titanium Pull Spray Superficial (TPSS) implant surface (Figure 1). Furthermore, the conometrical prosthetic joint with a switching platform [21,22] helps limiting the marginal bone level alteration and offers a better sealing to reduce bacterial colonization in respect to other connections [18,23].

### 2.3. Surgical Procedure

All patients underwent the same pre- and post-surgical protocol. The local anesthesia was induced by infiltration of 2 mL of Articaine 4% (40 mg/mL) and 1:100,000 Adrenalin. Full thickness mucoperiosteal flaps were raised by means of an intrasulcular and two vertical incisions.

**Figure 2:**
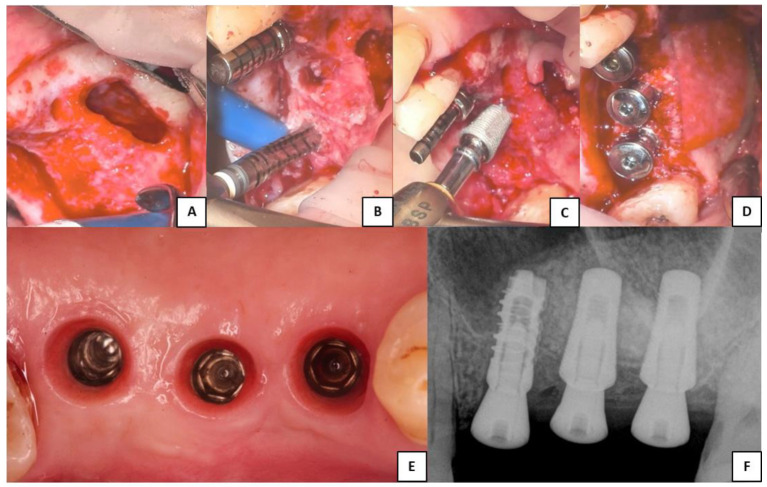
Clinical phases of the lateral sinus augmentation procedure performed. (**A**) Schneiderian membrane detached from the sinusal walls and elevated. (**B**) Bone grafting and implant site drilling preparation. (**C**) Two Sinus-Plant implants were positioned in very low basal bone height (about 2 mm), whereas one cylindrical implant (which is not part of the study) was inserted where the bone height was higher than 5 mm. (**D**) Covering of the lateral bony wall by a resorbable collagen membrane, before suturing of the flaps and the insertion of the healing abutments. (**E**) Soft tissue conditioning and removal of the healing abutments. (**F**) Periapical RX scan performed after the implants’ healing period (the two Sinus-Plant implants on the right).

Figure 2 was chosen as representative of the lateral surgeries performed and to show the only implant different from Sinus-Plant ones, which was inserted as part of a bridge. In this particular case, where three contiguous implants with different RBH were present and for which a different surgical approach should be suggested, the surgeon decided to perform a lateral approach to insert all the implants.

The lateral window technique for the sinus augmentation was performed by using a 1:200,000 rotary handpiece and a 2 mm round diamond bur [24]. Then, the bony window was overturned into the sinus and the membrane was elevated. The integrity of the Schneiderian membrane was ensured by covering it with a resorbable membrane (Cytoplast, RTM Collagen Membrane), and, later, the bone substitute was inserted into the sinus. As listed in Table 3, different grafting materials were used in this study, and another resorbable membrane (Cytoplast) covered the lateral bony wall. At the end, the flaps were sutured.

In both the sinus augmentation techniques, a 2 mm pilot drill was used to perforate the alveolar crest and then, twist drills of increasing diameters of the Sinus Oralplant drill kit were employed to enlarge the diameter of the osteotomy. Even the osteotomy drills presented a bunt apex to preserve the Schneiderian membrane during the preparation procedure. In performing the elevation techniques, the surgeon preferred to not involve the use of osteotomes that can fracture the internal cortex, but he rather used techniques that consumed the internal cortex, such as Ferdinando Cosci’s technique [25] or the VERSAH osseodensifying approach, conceived in 2013 by Huwais S. [26].

**Figure 3:**
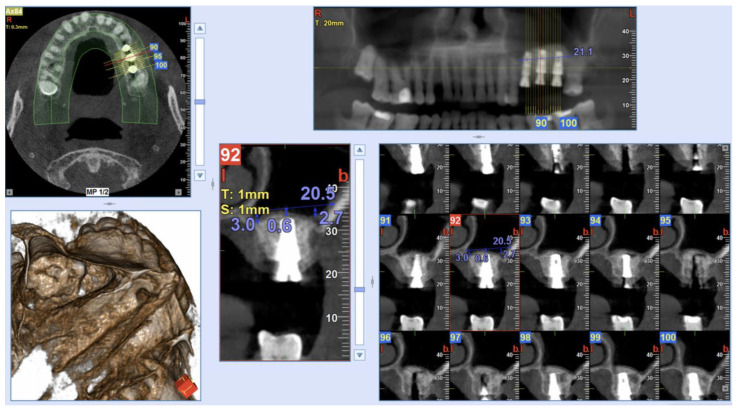
Post-operative CBCT scan after the surgical procedure and implant positioning. Measurements were taken on implants placed in 26 and 27 implant sites (Sinus-Plant implants).

The Insertion Torque values (IT) were measured during the implant placement with a surgical dynamometric wrench ratchet, according to the implant system protocol [27,28]. In all surgeries, IT values >25 Ncm were found, and so a one-stage sinus augmentation has been performed. As a result, 3-, 4-, 5- and 6-mm length prefabricated healing abutments were placed. The dimension of such abutments was selected according to the thickness of the keratinized mucosa. Figure 3 shows a Cone-Beam Computed Tomography (CBCT) scan (NewTom Giano HR, Cefla, Imola, Italy) performed immediately post-surgery. Sutures were removed 10 days after surgery.

All the patients were advised to follow a soft diet for at least 4 weeks and to undergo antimicrobial prophylaxis with Amoxicillin 825 mg and Clavulanic Acid 125 mg (Augmentin, GlaxoSmithKline, England) twice daily for 6 days, starting on the morning of the surgery. Ketoprofen 80 mg (OKI, Dompé, Italy) was prescribed as analgesic, twice daily for 3–4 days, as needed. Sixty days after surgery, periapical x-rays were taken only to check if the osseointegration process has become established; then, provisional or definitive crowns were placed 6 months after the procedure. 6 months after surgery, the technicians created temporary bridges, cemented in the case of 2 or more implants (24 patients and 25 bridges), with silicone impression material with pick-up abutments on prefabricated cylindrical abutments screwed to 25 N. After 7 months from the procedure, the abutments were tightened (40 N), and, finally, the dental impressions were recorded using a custom trays and polyvinylsiloxane impression material. The definitive metal (cobalt-chrome)/ceramic crowns were fixed with provisional cement (TempBond: Kerr Corp. Orange, CA, USA) approximately 8 months after the implant insertion. For multiple contiguous implants, the crowns were fabricated from metal-ceramic and then splinted. The single prosthetic crowns were made in lithium-disilicate material. All final restorations were placed in full occlusion and first checked with a 20-micron thickness articulated paper and then, with an 8-micron thickness Shimstock foil (Almore International; Portland, OR, USA). All the patients were recalled for a professional cleaning treatment by a dental hygienist every 3 months.

Study data were recorded before surgery, immediately after surgery, and after 12 and 36 months during the maintenance regimen, using the paralleling technique to take periapical digital x-rays. The peri-implant bone level changes were considered as variation of the distance between the implant-abutment junction and the highest coronal point of the supporting bone. Measurements were recorded in the mesial and distal areas of the implant and then, averaged.

### 2.4. Tomographic Examination

All measurements were performed in random order by two independent examiners, according to a previously described technique by Kawakami et al. [29,30,31,32,33,34].

**Figure 4:**
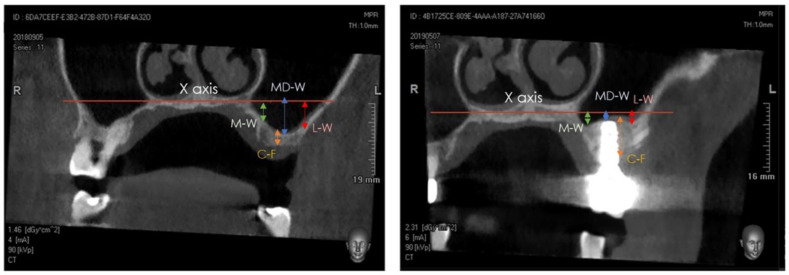
Detail of tomographic measurements before surgery (**left**) and after implant positioning (**right**). In both images, measurements were taken at the medial (sinus floor level at M-W), middle (sinus floor level at MD-W), and lateral (sinus floor level at L-W) level.

**Figure 5:**
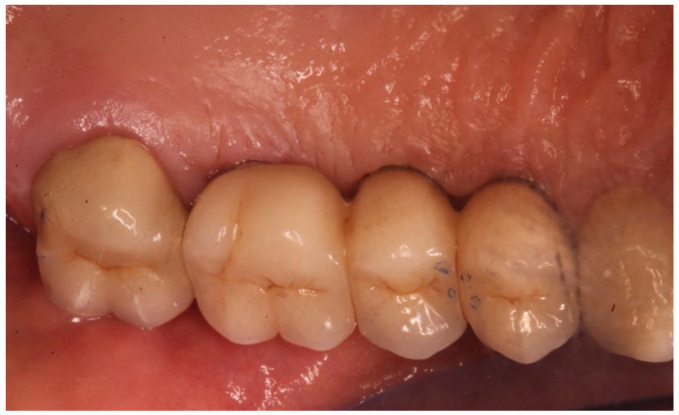
Detail of the prosthetic finalization after the occlusion check.

A Cone-Beam Computed Tomography (CBCT) scan was performed at T0 (pre-surgery), at T1 (immediately post-surgery), and at T2 (after 1 year). The distance between the center of the bone crest and the base of the maxillary sinus floor (C-F) on the coronal view were determined at T0 and T2, using the NNT software package (NewTom, Cefla, Imola, Italy). Furthermore, the distance between the highest position of the bone/xenograft tissue and a horizontal reference line, drawn following the floor of the nose (X axis), was also measured on the coronal view at T1 and T2, considering the following anatomical landmarks (Figure 4 and Figure 5) [29,30,31,32,33,34]:

(a) medial sinus floor level (M-W);
(b) middle sinus floor level (MD-W);
(c) lateral sinus floor level (L-W).

### 2.5. Statistical Analysis

The sample size calculation has been performed by the One-Way Analysis of Variance (ANOVA)-repeated measures [effect size: 0.16, α err: 0.05; power (1-β): 80%, N:4]. The minimum number of implants needed for a statistically significant output was 56 units, whereas a total of 69 implants was considered for the present retrospective study. Statistical analysis of the peri-implant bone level changes was performed by the ANOVA test, followed by the Tukey HSD multiple comparison test for post hoc comparison. After evaluating that the data for C-F were normally distributed (Kolmogorov-Smirnov test), the mean values at T0 and T2 were calculated and then, compared using a paired *t*-test. A multiple linear regression approach was used to assess the significance of the study variables.

Data for the maxillary sinus floor level (at M-W, at MD-W and at L-W) were not normally distributed. Therefore, after averaging T1 and T2 values, the data sets were compared using non-parametric Wilcoxon Signed Rank tests. Values were expressed as mean ± SD. Statistical evaluation was performed by the SPSS software package (IBM, Armonk, NY, USA). The α level was set at 0.05 for all tests and *p*-values < 0.05 were considered statistically significant. 

## 3. Results

The enrollment period of the patients lasted 22 months, from September 2016 to July 2018. 54 patients were enrolled and treated using 69 implants.

All the patients presented a type IV bone density, and all implants were characterized by an IT value >25 Ncm, so it was possible to proceed with immediate prefabricated healing abutments, without the need for a submerged healing period.

### 3.1. Implant Survival Rate

One implant fixture was lost after 14 months in a smoker patient, owing to the implant mobility after loading. The remaining implants (68) arrived at the end of the 3-year follow-up. A total of 1 implant was lost (implant size 11 × 4.5 mm), with a cumulative survival rate (CSR) of 98.55% after 36 months.

### 3.2. Surgical Complications

A total of 2 surgical complications were reported, one with sinus lateral approach and the other with the transcrestal approach. Both the small membrane perforations were treated by covering them with a bioresorbable membrane (Cytoplast, RTM Collagen Membrane). Subsequently, at a follow-up of 36 months, no implant failures were reported for either patient.

No fractures or loosening of abutments or prosthetic screws were reported during the study.

### 3.3. Marginal Bone Level Changes

Readable periapical radiographs were obtained for 54 patients after 36 months. At the end of the observational period, the bone level was, on average, 0.78 ± 1.06 mm below the implant apex (Table 4). A total of four implants showed bone levels exceeding 3 mm.

### 3.4. Cone-Beam Computed Tomography Assessment

**Figure 6:**
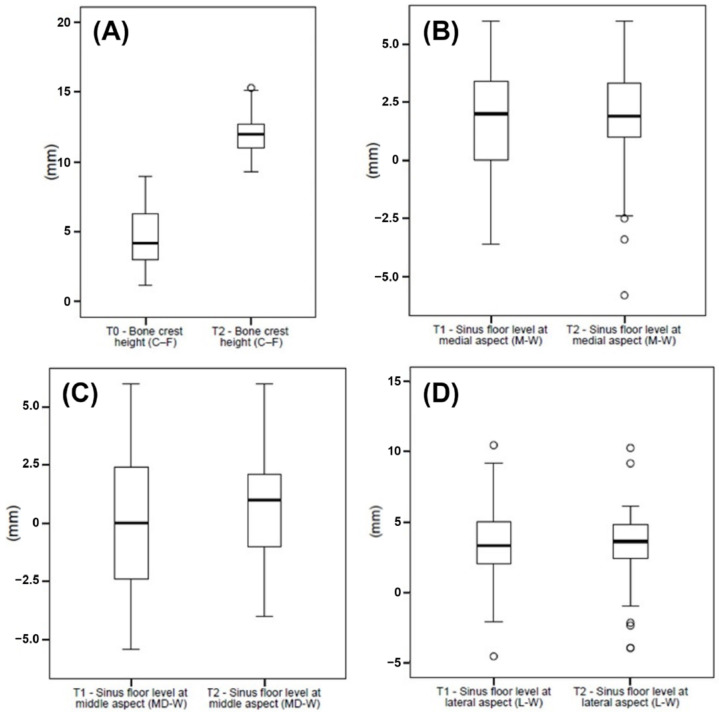
(**A**) Data of the bone crest height (C-F) collected at T0 and T1, and (**B**) data of the sinus floor level at medial (M-W), (**C**) middle (MD-W), and (**D**) lateral (L-W) aspects collected at T1 and T2, graphically summarized in boxplots. The central line in the boxes represents the median, and the boxes represent the middle distribution of values (ranging from the 25th to the 75th percentile). The whiskers show the extent of the data. Empty circles represent outliers.

The CBCT scans were obtained at T0, immediately post-surgery (T1), and at the 1-year follow-up (T2). The mean bone crest height (C-F) was 4.6 ± 2.0 at T0 and 12.1 ± 1.4 at T2 (*p* < 0.001). The sinus floor levels at M-W were 1.9 ± 2.4 at T1 and 1.7 ± 2.6 at T2 (*p* > 0.05). The measurements for the sinus floor level at MD-W were −0.1 ± 2.7 at T1 and 0.7 ± 2.4 at T2 (*p* = 0.004), whereas the sinus floor levels at L-W were 3.1 ± 3.0 at T1 and 3.1 ± 3.0 at T2 (*p* > 0.05) (Figure 6, Table 5 and Table 6).

## 4. Discussion

The decision workflow for the implant rehabilitation of the atrophic maxillary region depends on anatomic factors, such as bone quality and quantity [12]. Traditional approaches for inserting an implant of at least 10 mm in length include the use of the transcrestal sinus lift, in cases of 5–8 mm of RBH, and the lateral wall technique, in cases of less than 5 mm of RBH. From the literature, it has been shown that the simultaneous implant insertion (one-stage protocol) is only possible with a RBH of at least 3 mm, whereas, in cases of lower values, a second surgery is required to insert the implant after the end of a healing period, so it is compatible with the bone formation [17]. However, Mardinger et al. in 2007 suggested that it was possible to proceed with the one-stage protocol also with 1–3 mm of RBH by carefully planning the case and the clinical procedure [35].

On these bases, the objective of the present retrospective study was to evaluate the 3-year follow-up clinical and radiographic results of the use of an innovative reverse troncoconical implant design [18], which could allow to obtain at the same time (one-stage approach) a maxillary sinus augmentation procedure through transcrestal or lateral approaches and the implant insertion, even in challenging scenarios, such as with bone heights close to 2 mm. All sites included in this study were classified as type IV, according to Lekholm & Zarb [36]. Triches et al. recently evidenced the importance of preoperative identification of bone type IV in order to avoid the risk of early implant failures due to the sub-preparation of the implant site, the bicortical anchorage, the submerged positioning of the fixture, the need of a two-stage protocol, the immediate or early loading, and the use of implants with surface treatments [37].

All implants included in this retrospective study were characterized by IT values > 25 Ncm, and, therefore, despite the poor bone quality of the residual bone, the immediate insertion of the healing screw was allowed. The IT value is a key element for the achievement of osseointegration, and it influences the decision workflow of the implant loading protocols [38,39]. Indeed, the presence of an adequate primary stability enables to avoid micromovements >150 μm, which have a detrimental effect on bone formation around the implant surface [40]. Furthermore, the IT value is a mechanical parameter that is mainly influenced by the surgical procedure, the implant design, and the bone quality at the implant site [41]. Considering that all sites were characterized by a low quality of the bone, the good primary stability of these implants was a direct consequence of the Sinus-Plant implant design [18] that led to the osseodensification of the bone during the insertion procedure protocol [27,28]. In addition to the use of this type of implants, the one-stage approach allowed to stabilize the implant up to bone heights close to 2 mm, reducing the morbidity and giving the main advantage of reducing the total time for the final resolution of the case. In fact, the sinus lift procedure, the implant insertion, and the healing screw connection could be completed in a single time.

One major limitation of this study is that one implant, different from Sinus-Plant in shape and in the type of bone where inserted, was included to hold a bridge in place together with other two Sinus-Plant implants. Given the retrospective nature of the present study, unfortunately, it is not possible to consider and discuss the relative importance of this implant in the long-term implant stability results, even though it is plausible that it could contribute to the stability of the bridge.

The heterogenicity of the biomaterials and bone grafts used represented another limitation of the present study, although the key rationale of the study was to investigate the effect of the self-condensing dental implant design, simultaneously associated with a regenerative sinus lift surgical approach. However, the different bone substitutes did not affect the implant success parameters or produce significant differences in marginal bone loss over time. In particular, the height of the bone crest at the end of the surgeries significantly increased, with a mean of 12.1 ± 1.4 mm, when a first average of 4.6 ± 2.0 mm was measured before the surgical procedures. Consequently, the average bone height gained was 7.5 mm. The calculated bone gain, associated to the sinus augmentation procedure and achieved through the transcrestal and lateral sinus lift procedures, was comparable to data from studies that considered only the lateral approach (Sánchez-Recio et al. [42] measured an average bone gain of 7.2 mm).

In addition, in literature it was showed that the traditional transcrestal approach allowed a sinus floor elevation up to 5 mm in order to preserve the membrane integrity [43]. Accordingly, another important advantage of the Sinus-Plant design is the titanium lapping surface at the rounded apex, which allowed to preserve the Schneiderian membrane integrity, despite the great sinus elevation. Only two cases (2.89%) of small membrane perforations occurred, but they were immediately covered with a resorbable membrane. In this regard, it is important to highlight that membrane perforations have been reported in literature as a very frequent occurrence that can happen in the 25% of sinus lift procedures. These data are significantly higher in respect to our percentages [17]. Despite the literature supporting more bone formation when a collagen membrane was applied on a sinus mucosal perforation, it should be taken into account that there are also some researchers opposing the absolute need of such a procedure to enhance new bone formation and speculating that it could jeopardize the healing of the mucosal rupture [44,45].

A recent review of Lundgren et al. evidenced that the first year of function after sinus floor elevation is crucial for implant survival, because the failure rate is higher during this period [17]. In this case, only one implant was lost for early osseointegration failure within the sixth month. After 14 months, a second implant was lost due to the implant mobility starting from the fourth month after loading. Even though both implants failed within the third year of follow-up, they reached an optimal primary stability (IT > 30 Ncm) after being placed in concomitance with lateral sinus elevation. Interestingly, they were both inserted in smoker patients (≤10 cigarettes/day) who also referred bruxism and clenching parafunctions. These factors turned out to be crucial because smoking, as also reported by other authors [18], is claimed to be the main significant risk factor for early implant failures; additionally, bruxism favors mechanical complications without increasing the risk for biological ones [46,47]. However, there are also other factors affecting implant or sinus augmentation failures, such as the appropriate experience and training of the operator [48]. In this retrospective study, the cumulative survival rate of the implants reached the 100% and the 98.55% at baseline and after 3 years of follow-up, respectively, with only one implant failure. These values were significantly higher in respect to that reported by Mordenfeld et al. [49] (91.7%, 90.7%, 86.1%, and 86.1% at the placement, at 1 year post-loading, after 1–2 years, and 2–3 years, respectively), and by Pjetursson et al. [50], who reported an annual implant failure rate of 3.48% by using the lateral approach, and a 3-year survival rate of 90.1%, but reached 98.3% with the use of treated surface implants and a membrane to cover the lateral window. In a more recent retrospective study, Park et al. [2] reported an acceptable survival (95% and 85% at 10- and 20-year follow-up, respectively) of implants placed in residual bone height < 3 mm using a similar lateral sinus lift approach. Although the follow-up period was longer than 3 years, the insertion of Sinus-Plant implants with a one-stage technique reported a higher cumulative survival rate just after 36 months, confirming the advantage to reach a good primary stability, even in challenging bone height conditions.

As regards the Sinus-Plant implants inserted through the transcrestal approach, no losses were reported in the 3 years. These results seemed noteworthy, because they were higher in respect to the 98% (95% CI 96% to 100%) survival rate reported by Yan et al. in 2017 [51]. In addition, the marginal bone loss rates recorded by the same authors were higher than these levels found after 6, 12, and 36 months. Furthermore, the changing levels of the marginal bone after 3 years of follow-up (0.78 ± 1.06 mm) showed results that were almost halved if compared to that of Si et al. [52] in a transcrestal sinus lift with and without bone graft (1.33 ± 0.46 and 1.38 ± 0.23, respectively). Similarly, the values reported in the present study were also lower in respect to those of Mordenfeld et al. [49], who performed the sinus augmentation through the lateral wall technique. The low marginal bone loss values could be attributed to the conometric switching platform connection of this type of implants that ensured a better antibacterial sealing, which is a well-known key factor in bone loss, in respect to other connections [23,53].

## 5. Conclusions

The process of osseodensification occurring with the insertion of Sinus-Plant implants was related to the self-condensing tronco-conical implant design, which also ensured a sufficient clinical primary stability for an immediate positioning of the fixture in sinus grafting procedures (one-stage approach), even with a bone height close to 2 mm and a low bone quality, as an alternative to a two-stage approach. The implants macromorphology allowed to reach a higher amount of sinus elevation, without increasing the risk of membrane perforations. Moreover, the marginal bone loss was minimized, thanks to the conometric prosthetic connection, in order to ensure a better gingival maintenance and a reduced patient morbidity.

Within the limitations of this retrospective study, the association of this novel implant design with a one-stage sinus lift approach showed a high survival rate and also a low marginal bone loss after 3 years of follow-up. Further experimental studies with a higher number of cases and a longer follow-up time will be necessary in the future in order to confirm the findings of the present investigation.

## Figures and Tables

**Table 1:** Characteristics of the study population.

Population	*N*
Age (mean)	61.6 ± 11.2 years
Patients (Gender)	54 (26 female; 28 male)
RBH	4.51 ± 2.14 mm
Smokers (≤10 cig/die)	6/54 patients
Surgical approach	23 Transcrestal 31 Lateral

**Table 2:** Implant distribution according to the implant size and position.

Implant Size and Position	9 mm × 4.5 mm	10 mm × 4.5 mm	11 mm × 4.5 mm	*p* Value
Molars	1 (1.45%)	20 (28.98%)	43 (62.32%)	*p* > 0.05
Premolars	-	2 (2.90%)	3 (4.34%)	*p* > 0.05
Total	1 (1.45%)	22 (31.88%)	46 (66.67%)	-

**Table 3:** Implant distribution according to the bone graft.

Bone Graft	Surgical Sites
Kyeron	15
Mix Tutobone, Skeligraft (50:50)	10
Mix Autologous graft, Tutobone, Bioresorb (20:40:40)	9
Putty Osteobiol	8
Autologous graft	7
Mix Tutobone, Bioresorb (70:30)	5
Antemi	3
Mix Putty Osteobiol, Tutobone, Skeligraft (60:20:20)	3
Fibrin sponge	3
Mix Apathos Osteobiol, Tutobone, Bioresorb (40:40:20)	2
Mix Bioresorb, Putty Osteobiol 30:70	1
Mix Autologous graft, Skeligraft 80:20	1
Mix Tutobone, Putty Osteobiol 50:50	1
Mix Autologous graft, Gel 40 Osteobiol, Bioresorb 20:40:40	1
Mix Autologous graft, Putty Osteobiol 10:90	1
Mix Apathos, Gel 40 Osteobiol	1
Mix Autologous graft, Bioresorb 50:50	1
Total	54

**Table 4:** Changing levels of the marginal bone around the implant positioned in the grafted sinus.

	6 Months	12 Months	36 Months
Minimum	0.22	0.31	1.01
Maximum	0.90	0.93	3.06
Mean	0.53	0.78	1.70
Std. Deviation	0.06	0.06	0.16

*p* > 0.05 ANOVA and Tukey’s post-hoc test.

**Table 5:** Mean values and standard deviations (SD) of the distances between the bony crest center and sinus floor base (C-F) calculated at T0 and T2.

Timepoints	Bone Crest Height (C–F) (mm) ± SD
T0	4.6 ^b^ ± 2.0
T2	12.1 ^a^ ± 1.4

^a, b^ Same superscript letters indicate not statistically significant differences.

**Table 6:** Mean values and standard deviations (SD) for the sinus floor levels at the medial (M-W), middle (MD-W), and lateral (L-W) aspects calculated at T1 and T2.

	Sinus Floor Level at Medial Aspect (M-W) (mm) ± SD	Sinus Floor Level at Middle Aspect (MD-W) (mm) ± SD	Sinus Floor Level at Lateral Aspect (L-W) (mm) ± SD
T1	1.9 ^a^ ± 2.4	−0.1 ^b^ ± 2.7	3.1 ^a^ ± 3.0
T2	1.7 ^a^ ± 2.6	0.7 ^a^ ± 2.4	3.1 ^a^ ± 3.0

^a, b^ Same superscript letters indicate not statistically significant differences.

## Data Availability

All experimental data to support the findings of this study are available contacting the corresponding author upon request.

## References

[1] Seong W.J., Barczak M., Jung J., Basu S., Olin P.S., Conrad H.J. (2013). Prevalence of sinus augmentation associated with maxillary posterior implants. J. Oral Implantol..

[2] Park W.B., Kang K.L., Han J.Y. (2019). Factors influencing long-term survival rates of implants placed simultaneously with lateral maxillary sinus floor augmentation: A 6- to 20-year retrospective study. Clin. Oral Implant. Res..

[3] Lemos C.A.A., Ferro-Alves M.L., Okamoto R., Mendonça M.R., Pellizzer E.P. (2016). Short Dental Implants versus Standard Dental Implants Placed in the Posterior Jaws: A Systematic Review and Meta-Analysis. J. Dent..

[4] Villarinho E.A., Cervieri A., Shinkai R.S.A., Grossi M.L., Teixeira E.R. (2015). The Effect of a Positioning Index on the Biomechanical Stability of Tapered Implant-Abutment Connections. J. Oral Implantol..

[5] Tatum H. (1986). Maxillary and Sinus Implant Reconstructions. Dent. Clin. North Am..

[6] Krasny K., Krasny M., Kamiński A. (2015). Two-stage closed sinus lift: A new surgical technique for maxillary sinus floor augmentation. Cell Tissue Bank..

[7] Pesce P., Menini M., Canullo L., Khijmatgar S., Modenese L., Gallifante G., Del Fabbro M. (2021). Radiographic and Histomorphometric Evaluation of Biomaterials Used for Lateral Sinus Augmentation: A Systematic Review on the Effect of Residual Bone Height and Vertical Graft Size on New Bone Formation and Graft Shrinkage. J. Clin. Med..

[8] Al-Moraissi E., Alhajj W.A., Al-Qadhi G., Christidis N. (2022). Bone Graft Osseous Changes After Maxillary Sinus Floor Augmentation: A Systematic Review. J. Oral Implantol..

[9] Abbott J.R., Marino V., Bartold P.M. (2014). Human Cadaveric Histomorphological and Metallurgical Analysis of Dental Implants Following 12.5 Years of Service. Clin. Oral Implant. Res..

[10] Ashman A. (2000). Postextraction Ridge Preservation Using a Synthetic Alloplast. Implant. Dent..

[11] Tumedei M., Piattelli A., Degidi M., Mangano C., Iezzi G. (2020). A Narrative Review of the Histological and Histomorphometrical Evaluation of the Peri-Implant Bone in Loaded and Unloaded Dental Implants. A 30-Year Experience (1988-2018). Int. J. Environ. Res. Public Health.

[12] Carreño Carreño J., Aguilar-Salvatierra A., Gómez-Moreno G., García Carreño E.M., Menéndez López-Mateos M.L., Perrotti V., Piattelli A., Calvo-Guirado J.L., Menéndez-Núñez M. (2016). Update of Surgical Techniques for Maxillary Sinus Augmentation: A Systematic Literature Review. Implant. Dent..

[13] Avila-Ortiz G., Wang H.-L., Galindo-Moreno P., Misch C.E., Rudek I., Neiva R. (2012). Influence of Lateral Window Dimensions on Vital Bone Formation Following Maxillary Sinus Augmentation. Int. J. Oral Maxillofac. Implant..

[14] Gonzalez S., Tuan M.-C., Ahn K.M., Nowzari H. (2014). Crestal Approach for Maxillary Sinus Augmentation in Patients with ≤4 Mm of Residual Alveolar Bone. Clin. Implant. Dent. Relat. Res..

[15] Monje A., Ravidà A., Wang H.L., Helms J.A., Brunski J.B. (2019). Relationship Between Primary/Mechanical and Secondary/Biological Implant Stability. Int. J. Oral Maxillofac. Implant..

[16] Borges F.L., Dias R.O., Piattelli A., Onuma T., Gouveia Cardoso L.A., Salomão M., Scarano A., Ayub E., Shibli J.A. (2011). Simultaneous Sinus Membrane Elevation and Dental Implant Placement without Bone Graft: A 6-Month Follow-up Study. J. Periodontol..

[17] Lundgren S., Cricchio G., Hallman M., Jungner M., Rasmusson L., Sennerby L. (2017). Sinus Floor Elevation Procedures to Enable Implant Placement and Integration: Techniques, Biological Aspects and Clinical Outcomes. Periodontol. 2000.

[18] Comuzzi L., Iezzi G., Lucchese A., Di Pietro N., Balice P., D’Arcangelo C., Piattelli A., Tumedei M. (2022). Mechanical Behaviour and Primary Stability of a Self-Condensing Implant: A Laboratory Critical Simulation of a Severe Maxillary Atrophy on Polyurethane Lamina. Appl. Sci..

[19] Falco A., Berardini M., Trisi P. (2018). Correlation Between Implant Geometry, Implant Surface, Insertion Torque, and Primary Stability: In Vitro Biomechanical Analysis. Int. J. Oral Maxillofac. Implant..

[20] Gehrke S.A., Pérez-Díaz L., Mazón P., De Aza P.N. (2019). Biomechanical Effects of a New Macrogeometry Design of Dental Implants: An In Vitro Experimental Analysis. J. Funct. Biomater..

[21] Iezzi G., Iaculli F., Calcaterra R., Piattelli A., Di Girolamo M., Baggi L. (2017). Histological and Histomorphometrical Analysis on a Loaded Implant With Platform-Switching and Conical Connection: A Case Report. J. Oral Implantol..

[22] Raj H.K., Mohan T.K., Kattimani V., Sreerama R., Ramya Y., Inampudi C.K. (2022). Evaluation of Immediately Loaded Parallel Conical Connection Implants with Platform Switch in the Maxillary Esthetic Zone: A Prospective Clinical Study. J. Contemp. Dent. Pract..

[23] D’Ercole S., Scarano A., Perrotti V., Mulatinho J., Piattelli A., Iezzi G., Tripodi D. (2014). Implants with Internal Hexagon and Conical Implant-Abutment Connections: An in Vitro Study of the Bacterial Contamination. J. Oral Implantol..

[24] Wallace S.S., Tarnow D.P., Froum S.J., Cho S.C., Zadeh H.H., Stoupel J., Del Fabbro M., Testori T. (2012). Maxillary sinus elevation by lateral window approach: Evolution of technology and technique. J. Evid. Based Dent. Pract..

[25] Esposito M., Cannizzaro G., Barausse C., Cosci F., Soardi E., Felice P. (2014). Cosci versus Summers technique for crestal sinus lift: 3-year results from a randomised controlled trial. Eur. J. Oral Implantol..

[26] Huwais S., Meyer E.G. (2017). A Novel Osseous Densification Approach in Implant Osteotomy Preparation to Increase Biomechanical Primary Stability, Bone Mineral Density, and Bone-to-Implant Contact. Int. J. Oral Maxillofac. Implant..

[27] Oralplant—Produttori Di Implantologia. https://www.oralplant.ch/index.php/it/.

[28] Carinci F., Pezzetti F., Volinia S., Francioso F., Arcelli D., Marchesini J., Scapoli L., Piattelli A. (2003). Analysis of Osteoblast-like MG63 Cells’ Response to a Rough Implant Surface by Means of DNA Microarray. J. Oral Implantol..

[29] Kawakami S., Lang N.P., Iida T., Ferri M., Apaza Alccayhuaman K.A., Botticelli D. (2018). Influence of the Position of the Antrostomy in Sinus Floor Elevation Assessed with Cone-Beam Computed Tomography: A Randomized Clinical Trial. J. Investig. Clin. Dent..

[30] Kawakami S., Lang N., Ferri M., Alccayhuaman K., Botticelli D. (2019). Influence of the Height of the Antrostomy in Sinus Floor Elevation Assessed by Cone Beam Computed Tomography: A Randomized Clinical Trial. Int. J. Oral Maxillofac. Implant..

[31] Kawakami S., Botticelli D., Nakajima Y., Sakuma S., Baba S. (2019). Anatomical Analyses for Maxillary Sinus Floor Augmentation with a Lateral Approach: A Cone Beam Computed Tomography Study. Ann. Anat. Anat. Anz..

[32] Imai H., Lang N., Ferri M., Hirota A., Alccayhuaman K. (2020). Tomographic Assessment on the Influence of the Use of a Collagen Membrane on Dimensional Variations to Protect the Antrostomy After Maxillary Sinus Floor Augmentation: A Randomized Clinical Trial. Int. J. Oral Maxillofac. Implant..

[33] Hirota A., Lang N.P., Ferri M., Fortich Mesa N., Apaza Alccayhuaman K.A., Botticelli D. (2019). Tomographic Evaluation of the Influence of the Placement of a Collagen Membrane Subjacent to the Sinus Mucosa during Maxillary Sinus Floor Augmentation: A Randomized Clinical Trial. Int. J. Implant. Dent..

[34] Sakuma S., Ferri M., Imai H., Fortich Mesa N., Blanco Victorio D.J., Apaza Alccayhuaman K.A., Botticelli D. (2020). Involvement of the Maxillary Sinus Ostium (MSO) in the Edematous Processes after Sinus Floor Augmentation: A Cone-Beam Computed Tomographic Study. Int. J. Implant. Dent..

[35] Mardinger O., Nissan J., Chaushu G. (2007). Sinus Floor Augmentation with Simultaneous Implant Placement in the Severely Atrophic Maxilla: Technical Problems and Complications. J. Periodontol..

[36] Lekholm U., Branemark P.I., Zarb G.A., Albrektsson T. (1985). Patient Selection and Preparation. Tissue Integrated Prostheses: Osseointegration in Clinical Dentistry.

[37] Triches D.F., Alonso F.R., Mezzomo L.A., Schneider D.R., Villarinho E.A., Rockenbach M.I., Teixeira E.R., Shinkai R.S. (2019). Relation between Insertion Torque and Tactile, Visual, and Rescaled Gray Value Measures of Bone Quality: A Cross-Sectional Clinical Study with Short Implants. Int. J. Implant. Dent..

[38] Calandriello R., Tomatis M., Rangert B. (2003). Immediate Functional Loading of Brånemark System Implants with Enhanced Initial Stability: A Prospective 1- to 2-Year Clinical and Radiographic Study. Clin. Implant. Dent. Relat. Res..

[39] Del Giudice R., Piattelli A., Grande N.-M., Cataneo E., Crispino A., Petrini M. (2019). Implant Insertion Torque Value in Immediate Loading: A Retrospective Study. Med. Oral Patol. Oral Cir. Bucal..

[40] Szmukler-Moncler S., Piattelli A., Favero G.A., Dubruille J.H. (2000). Considerations Preliminary to the Application of Early and Immediate Loading Protocols in Dental Implantology. Clin. Oral Implant. Res..

[41] Bayarchimeg D., Namgoong H., Kim B.K., Kim M.D., Kim S., Kim T.-I., Seol Y.J., Lee Y.M., Ku Y., Rhyu I.-C. (2013). Evaluation of the Correlation between Insertion Torque and Primary Stability of Dental Implants Using a Block Bone Test. J. Periodontal. Implant. Sci..

[42] Sánchez-Recio C., Peñarrocha-Diago M., Peñarrocha-Diago M., Peñarrocha-Oltra D. (2010). Maxillary Sinus Lift Performed Using Ultrasound. Evaluation of 21 Patients. Med. Oral Patol. Oral Cir. Bucal..

[43] Engelke W., Deckwer I. (1997). Endoscopically Controlled Sinus Floor Augmentation. A Preliminary Report. Clin. Oral Implant. Res..

[44] Favero V., Lang N.P., Canullo L., Urbizo Velez J., Bengazi F., Botticelli D. (2016). Sinus floor elevation outcomes following perforation of the Schneiderian membrane. An experimental study in sheep. Clin. Oral Implant. Res..

[45] Monje A., Diaz K.T., Aranda L., Insua A., Garcia-Nogales A., Wang H.-L. (2016). Schneiderian Membrane Thickness and Clinical Implications for Sinus Augmentation: A Systematic Review and Meta-Regression Analyses. J. Periodontol..

[46] Manfredini D., Poggio C.E., Lobbezoo F. (2014). Is Bruxism a Risk Factor for Dental Implants? A Systematic Review of the Literature. Clin. Implant. Dent. Relat. Res..

[47] Manzano G., Montero J., Martín-Vallejo J., Del Fabbro M., Bravo M., Testori T. (2016). Risk Factors in Early Implant Failure: A Meta-Analysis. Implant. Dent..

[48] Pinchi V., Varvara G., Pradella F., Focardi M., Donati M.D., Norelli G. (2014). Analysis of Professional Malpractice Claims in Implant Dentistry in Italy from Insurance Company Technical Reports, 2006 to 2010. Int. J. Oral Maxillofac. Implant..

[49] Mordenfeld A., Albrektsson T., Hallman M. (2014). A 10-Year Clinical and Radiographic Study of Implants Placed after Maxillary Sinus Floor Augmentation with an 80:20 Mixture of Deproteinized Bovine Bone and Autogenous Bone. Clin. Implant. Dent. Relat. Res..

[50] Pjetursson B.E., Tan W.C., Zwahlen M., Lang N.P. (2008). A Systematic Review of the Success of Sinus Floor Elevation and Survival of Implants Inserted in Combination with Sinus Floor Elevation. J. Clin. Periodontol..

[51] Yan M., Liu R., Bai S., Wang M., Xia H., Chen J. (2018). Transalveolar Sinus Floor Lift without Bone Grafting in Atrophic Maxilla: A Meta-Analysis. Sci. Rep..

[52] Si M., Zhuang L., Gu Y.-X., Mo J., Qiao S., Lai H.-C. (2013). Osteotome Sinus Floor Elevation with or without Grafting: A 3-Year Randomized Controlled Clinical Trial. J. Clin. Periodontol..

[53] Hsu Y.-T., Lin G.-H., Wang H.-L. (2017). Effects of Platform-Switching on Peri-Implant Soft and Hard Tissue Outcomes: A Systematic Review and Meta-Analysis. Int. J. Oral Maxillofac. Implant..

